# Accurate evaluation of mode of delivery and labor progression with angle of progression: a prospective cross-sectional

**DOI:** 10.61622/rbgo/2025rbgo5

**Published:** 2025-03-17

**Authors:** Huong Lam Le, Huy Vu Quoc Nguyen, Tam Minh Le, Lam Hoang Vo

**Affiliations:** 1 Hue University Hue University of Medicine and Pharmacy Department of Obstetrics and Gynecology Hue Vietnam Department of Obstetrics and Gynecology, Hue University of Medicine and Pharmacy, Hue University, Hue, Vietnam.

**Keywords:** Labor stage, second, Pregnancy, Cesarean section, Delivery, obstetric, Ultrasonography

## Abstract

**Objective:**

To determine the validity of the angle of progression (AoP) in predicting delivery mode among women in the second stage of labor.

**Designs:**

This prospective cohort study was conducted at the Obstetrics and Gynecology unit (OBGYN) of two hospitals in Vietnam. Transperineal ultrasound was performed for each woman to measure the progression angle in the second phase of labor.

**data were collected from**

**Participants:**

A total of 725 women with singleton pregnancies with cephalic presentation at term

**Methods:**

Transperineal ultrasound was used to measure the angle of progression in the second labor phase and to identify the delivery method.

**Results:**

The rate of vaginal birth in women with an AoP ≥ 120° on transperineal ultrasound was 70.2%. The optimal cutoff point of AOP ≥122° with sensitivity and specificity for vaginal birth were 87.8% and 80.7%, respectively the area under the ROC curve of 0.887 (p<0.0001). The study's sample size was restricted owing to deficiencies in resources and time.

**Conclusion:**

The likelihood of achieving spontaneous vaginal delivery can be predicted by the angle of progression measured with transperineal intrapartum ultrasonography during the second stage of labor in women.

## Introduction

It is crucial to closely monitor the well-being of both the maternal and the fetus during the second stage of labor management and to remain vigilant for any possible complications.^(1)^ A comprehensive monitoring approach is imperative at this stage. Utilizing ultrasound technology to evaluate fetal head engagement is highly advisable, as it can significantly aid in predicting labor outcomes.^(2)^

Ultrasound assessment of fetal presentation, measurement of the angle of progression (AoP), head-perineum distance, head-pubic symphysis distance, fetal orientation, and midline angle are essential for accurately evaluating fetal position and engagement before determining the mode of delivery. This can help avoid unnecessary cesarean section indications. According to the International Society of Ultrasound in Obstetrics and Gynecology (ISUOG) guidelines for intrapartum ultrasound practice, the technique for measuring these parameters is straightforward.^(3)^ Manual examination during prenatal visits may be subjective, and accuracy depends on the examiner's experience. Studies have shown mistake rates ranging from 20% to 70%, with misdiagnosis potentially leading to an increased risk of trauma to both the fetus and the mother during instrumental deliveries or unnecessary cesarean section indications in some cases.^(4)^

Accurately determining the fetal position and engagement before choosing the delivery method requires ultrasound evaluation. This process helps to avoid unnecessary recommendations for cesarean section. During labor, the Dell classification determines the lowest part of the presenting fetal part at the level of the ischial spines or the largest diameter of the presenting part aligned with the maternal pelvic inlet.^(1,5)^ The examiner's experience also influences the assessment of fetal descent. Complications may arise, especially if the fetal head shows caput succedaneum or skull molding. Recent studies indicate that transperineal ultrasound can be used to assess fetal descent during labor objectively, providing clinicians with valuable information for predicting labor progress.^(6)^ Making informed decisions is crucial to ensure the safety of both the mother and the child, whether to proceed with vaginal delivery or opt for a cesarean section. Intrapartum ultrasound is increasingly recognized as an objective tool for monitoring labor progression.

A comprehensive, supportive approach to vaginal examination is required, and studies have suggested that ultrasound, with its non-invasive nature and wealth of information, be used throughout delivery. Numerous studies have shown that pelvic floor ultrasonography can be utilized to identify dystocia in labor and forecast the possibility of a vaginal delivery that will benefit. Therefore, we conducted a research project: Accurate evaluation of mode of delivery and labor progression with intrapartum transperineal ultrasound to determine the validity of the angle of progression (AoP) in predicting delivery mode among women in the second stage of labor.

## Methods

A prospective cohort study included singleton pregnancies undergoing second-stage labor monitoring at the Obstetrics and Gynecology unit (OBGYN) of Hue Center Hospital and Hue University of Hospital from January 2022 to November 2023. Singleton pregnancy, cephalic presentation, and gestational age from 37 weeks to 41 weeks 6 days calculated based on the last menstrual period or first-trimester ultrasound. Women in the second stage of labor begin when the cervix is fully dilated and ends with the birth of the baby. Absence of abnormal labor signs:

- Pelvic inlet: clinical examination reveals no prominent sacral promontory, palpable up to half of the ischial spines, both sacral spines palpable, and no abnormal pelvic deformities.
- Sign of fetal distress with type II and III of cardiotocography due to ACOG guideline.^(7)^

Acute fetal distress during labor. Previous uterine surgery scars including cesarean section, myomectomy… Internal medical conditions were incompatible with vaginal delivery. The sample size was calculated based on a formula:


$$n\geq \frac{Z_{1-\frac{\text{α}}{2}}^{2}\left(1-p\right)p}{d^{2}}=\frac{1.96^{2}\times 0.2\times \left(1-0.2\right)}{0.03^{2}}=683$$

With Z is the critical value of the standard normal distribution (with α = 0.05, Z = 1.96), the International Federation of Gynecology and Obstetrics (FIGO) recommends a cesarean section rate of about 20%; p = 0.2. The estimated margin of error d = 0.03. The minimum required sample size is 683. In reality, the research gathered data from 725 different cases. In assessing pregnant women in the second stage of labor, we conducted a thorough examination, including fundal height measurements, abdominal circumference, and determination of fetal position through Leopold's maneuver. We also performed fetal heart auscultation, evaluated uterine contractions, and conducted a vaginal examination to assess pelvic structure, cervical dilation, fetal position, presentation, engagement, and amnion status. Fetal heart monitoring was carried out using obstetric tracking, and we meticulously recorded and evaluated the factors and outcomes of the second stage of labor. During the second stage of labor, our healthcare providers focused on assessing complete cervical dilation and effacement, as well as evaluating the strength and frequency of uterine contractions and the fetal heart rate using CTG. We also examined fetal position, presentation, engagement, and skull molding and conducted intermittent auscultation (IA) of the fetal heart after each contraction for at least one minute every five minutes.

Each pregnant woman underwent transperineal ultrasound to assess the angle of progression (AoP) without uterine contraction or pushing. This procedure was carried out by qualified professionals, including radiologists specialized in ultrasound imaging or obstetricians with advanced training and certification in obstetric ultrasound. The process involved the woman emptying her bladder and assuming a supine lithotomy position. A sterile cover was applied to the convex probe, which was then inserted vertically between the labia to obtain a sagittal view from the symphysis pubis's longitudinal axis. This approach enabled accurate measurement of the AoP during complete cervical dilation and the second stage of labor, ensuring precise and reliable assessment.^(8)^ The sagittal-view image was captured, and the angle between the symphysis pubis line and the line connecting the lowest point of the symphysis pubis to the fetal head was measured. We also assessed the time and delivery type. Furthermore, we recorded the head-symphysis distance (HSD), midline angle (MLA), head-perineum distance (HPD), and fetal head direction.^(3)^

Data analyses were conducted utilizing the statistical software SPSS 20.0. Prism 9 was also used to analyze the receiver operating curve (ROC). Mean and standard deviation (SD) represented continuous data per their distribution. The Independent Samples T-Test compares the means of two independent groups to determine if there is a significant difference between them. Logistic regression analysis was used to control for potential confounding variables, while the AuROC and its associated 95% confidence interval (95% CI) were used to assess the prediction of the mode of delivery. We used the optimal cutoff value determined by the Youden Index method to evaluate the diagnostic efficacy. In addition, the sensitivity (Se) and specificity (Sp) were calculated. The Ethics Committee of Hue University approved this study 1243/ QD-DHH, 29th August 2022.

## Results

Overall, the study comprised 725 women. The transperineal ultrasound examination was performed with success in every eligible case. The baseline and obstetrical characteristics of the group are presented in table 1. The average age of the participants was 25.34 years. In the sample, 57.6% of the women were nulliparous, while 42.4% were multiparous. The average height of the pregnant was 153.89 ± 4.96 cm, and the average gestational of pregnancy was 38.7 ± 1.7 weeks. Of all the participants, 26.9% had ≤ 3 contractions/10 minutes, while 73.1% had >3 contractions. Fetal station: At engagement, 0 and +1 accounted for 37.9%, while −1 and 2 were 14.4%. Oxytocin was used in 23.4% of the cases. Meconium staining was observed in 11.9% of the cases. Vaginal delivery was reported in 87.5% of the cases, while cesarean section accounted for 12.5%. Most fetal weight was between 2500 and 3500 grams, and the average fetal weight was 3138 ± 427 grams.

**Table 1:** Features of demographic, intrapartum and labor outcomes

Characteristics		n(%)
Maternal age (±SD)		25.34 ± 4,22
Parity	Nulliparous	418(57.6)
	Multiparous	307(42.4)
Maternal height (±SD) (cm)		153.89 ± 4.96
Maternal weight (±SD) (kg)	End of pregnancy	65,36 ± 5,67
	Weight gain during pregnancy	12,05 ± 3,47
Gestation age (±SD) (week)		38.7 ± 1.7
Uterine contractions	< 3 contractions / 10 mins	195(26.9)
	≥ 3 contractions / 10 mins	530(73.1)
Amniotic fluid color	Clear	640(88.3)
	Colour	85(11.7)
Fetal station	-2	25(3.4)
	-1	80(11.0)
	0	275(37.9)
	+1	275(37.9)
	+2	70(9.8)
	+3	0(0)
Caput succedaneum	Yes	79(11.9)
	No	646(89.1)
Mode of delivery	Vaginal delivery	635(87.5)
	Cesarean section	90(12.5)
Birthweight	<2500 gram	10(0.6)
	2500 – 3500 gram	540(74.4)
	>3500 gram	175(24.1)
	±SD (gram)	3138 ± 427g

**Figure 1:**
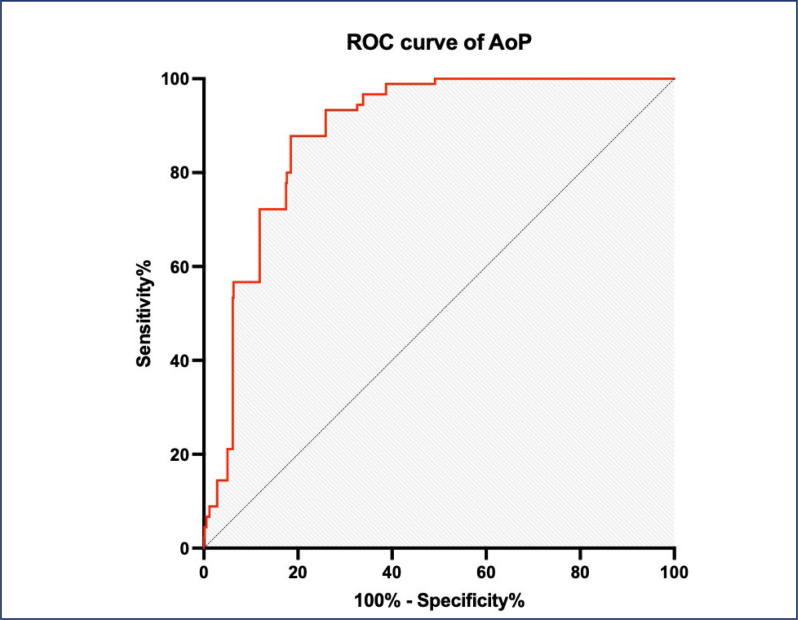
ROC curve analysis for the angle of progression. AuROC for the prediction of the mode of delivery was 0.867, 95% CI (0.86 - 0.91), P < 0.0001

According to the data presented in table 2, the median (Q25%-75%) of AoP through ultrasound measured 127.0 (119.0;131.0), with angles ≥120° accounted for 72.3%. The percentage of HPD < 45 mm was 83.5%, while the rate of <45 mm was 16.5%. The median of HPD was 38. Additionally, the median of HSD was 12 (11;12) mm, with the majority between 11-17 mm, accounting for 49.9% (Figure 1). Table 3 revealed that individuals with a birthweight of less than 3500 grams exhibited a mean AoP of 127.7 degrees, whereas those with a birthweight of 3500 grams or more had a mean AoP of 124.4 degrees. Moreover, multiparous individuals showed a mean AoP of 128.3 degrees, whereas primiparous individuals had a mean AoP of 126.5 degrees. The differences in birthweight (p = 0.015) and parity (p = 0.048) were statistically significant.

**Table 2:** Transperineal ultrasound parameters at the second stage of labor

Parameters		n(%)
AoP	< 120°	181(27.7)
	≥ 120°	544(72.3)
	Median (Q25%;Q75%)	127.0(119.0;131.0)
Head-perineum distance (HPD)	< 45 mm	605(83.5)
	> 45 mm	120(16.5)
	Median (Q25%;Q75%)	38(32;42)
Head-symphysis distance (HSD)	≥ 18 mm	32(4.4)
	17-11 mm	362(49.9)
	< 11 mm	331(45.7)
	Median (Q25%;Q75%)	12(11;12)
Midline angle (MLA)	MLA ≥ 45^0^	94(12.9)
	MLA < 45^0^	631(87.1)
	Median (Q25%;Q75%)	31(30;32)
Fetal head direction	< 0^0^	117(16.1)
	0^0^-30^0^	59(8.1)
	> 30^0^	549(75.8)

*AoP*, Angle of progression; HPD, Head-perineum distance; HSD, Head-symphysis distance; MLA, Midline angle

**Table 3:** Comparison of AoP by birthweight and parity characteristics

	Characteristics	AoP mean	SD	p-value
Birthweight	≥ 3500	124.4	9.4	0.015
	< 3500	127.7	7.9	
Parity	Primiparous	126.5	8.8	0.048
	Multiparous	128.3	7.2	

The AuROC of the transperineal ultrasound during the second stage of labor assessing vaginal delivery was 0.867 (95%CI 0.86-0.91) with p<0.0001. At the optimal cut-off point >122 degree, the sensitivity and specificity for predicting vaginal delivery were 87.8% and 80.1% (Youden Index: 68.6%), respectively.

## Discussion

This cross-sectional study examined the size of the AoP during the second stage of labor in fetuses with cephalic presentation and its association with spontaneous vaginal birth. The investigation's overall findings indicate that the progress angle directly influences the mode of delivery. This study established a direct correlation between the degree of the Angle of Progression (AoP) and the risk of a normal vaginal delivery. Furthermore, it was found that reducing the progression angle increased the likelihood of a cesarean section birth.

There is a notable correlation between the AoP and the delivery method. Numerous studies have shown that ultrasound evaluation of the fetus can objectively illustrate the contrast in vaginal progression between vaginal delivery and cesarean section. These findings are based on clinical applications of ultrasound in detecting AoP progression. A recent study was conducted with 88 nulliparous and multiparous women to evaluate the duration of the active phase of labor from the first to the second. The study found that successful vaginal delivery occurred in all cases where the AoP exceeded 120° during the second stage of labor.^(6)^ A new meta-analysis examined the diagnostic accuracy of AoP in predicting successful vaginal delivery in 887 pregnant women during the second stage of labor based on data from eight trials. The findings revealed that the test had a sensitivity of 80% (95% confidence interval [CI], 71-86%) and a specificity of 81% (95% CI, 72-88%). The area under the curve was 0.87, indicating a strong ability to detect spontaneous VD. The AoP range of 108°-119° had the highest sensitivity of 94% (95% CI, 88-97%), while the range of 141°-153° had the highest specificity of 82% (95% CI, 66-92%). Moreover, when the AoP varied between 141° and 158°, the likelihood of achieving effective vaginal delivery increased substantially from 22% to 87%.^(9)^ In subsequent studies aimed at predicting the delivery mode, the range of AoP values linked with successful vaginal delivery was found to be between 115° and 153.3°.^(10–13)^

Prior studies comparing 100 nulliparous and 71 multiparous pregnant women who were equivalent in gestational age and more than 39 weeks revealed that the likelihood of cesarean delivery was increased in nulliparous women with a narrow AoP of less than 95 degrees. Multiparous women exhibited a narrower AoP than nulliparous women before the onset of labor. Limited AoP in multiparous women does not appear to be associated with cesarean delivery, in contrast to nulliparous women, and the majority of multiparous women give birth vaginally. Our results, which differ from those of this study, demonstrated that in primigravida and multigravida women, lower AOP was associated with a more extended induction period and the second stage of labor.^(13)^ Tutschek et al.^(14)^ found that when the angle of progression during labor was 120° or more, the rate of vaginal delivery was 93%, which is higher than our study's rate. Gillor's research observed that patients who underwent cesarean section had a narrower angle of progression compared to those who delivered vaginally, and this difference was statistically significant.^(15)^ Similarly, Amin et al.^(16)^ reported that women with an angle of progression above 120° had a higher likelihood of delivering vaginally compared to those with an angle of progression below 120°. In our study, we found that patients with an angle of progression above 120° had a higher rate of vaginal delivery compared to those with an angle of progression below 120°, especially when the cervix was fully dilated, which is consistent with the findings of the Bamberg et al.^(17)^ study. This author compared ultrasound pelvic assessment with MRI assessment of engagement, and it found that an angle of progression of 120° corresponded to an engagement of 0 on MRI imaging. In our study, we used ultrasound pelvic assessment to determine the angle of progression at the time of complete cervical dilation, and we found that this measurement helped predict vaginal delivery.^(17)^ Malik and Singh^(18)^ found that an angle of progression of 116° or more during the late first and second phases of labor was a positive indicator for vaginal birth. Barbera et al.^(6,19)^ and Kalache et al.^(20)^ observed a consistent increase in the "angle of progression" during the second stage of all vaginal deliveries. They noted that all women with an angle of advancement more significant than 120° gave birth naturally. The cutoff value for patients who require a cesarean section was determined to be an angle of progression that is less than or equal to 108°.^(6,19,20)^

Research suggests that routine transperineal ultrasonography evaluation of the labor process is recommended. This simple imaging diagnostic technique takes less than 5 minutes, based on numerous studies. In our investigation, we recorded the fetal head direction, HPD, HSD, MLA, and angle of progression during the ultrasound pelvic evaluation in less than 3 minutes. The amount of time needed for ultrasonography assessment in our study was comparable to that of many other investigations. Many studies have shown that clinical obstetricians can easily acquire and apply transperineal ultrasound assessment to assess labor progress. Consequently, specialized maternity hospitals can effectively conduct training sessions and apply this novel ultrasound approach.

This is the first study to analyze the relationship between delivery outcomes and the angle of progression on transperineal sonography in Central Vietnam. Nonetheless, the application of this study could have been more extensive in certain aspects. First, the study's sample size is limited by resources and time, making various statistical analyses of the indicators examined insignificant. The research was carried out using a cross-sectional approach. It is essential to notice that research involving both control and study groups in an interventional trial will result in more trustworthy findings when examining how the angle of improvement affects delivery mode prediction. Additionally, the only angle of progression throughout the second labor stage was this study's focus.

## Conclusion

In singleton, term pregnancies with the cephalic presentation of the fetus, the ultrasound-based measurement of the AoP utilizing the transperineal ultrasound method at the onset of the second stage of labor at rest may enhance our capacity to predict a successful spontaneous vaginal delivery.
